# Detecting Drug–Target Interactions with Feature Similarity Fusion and Molecular Graphs

**DOI:** 10.3390/biology11070967

**Published:** 2022-06-27

**Authors:** Xiaoli Lin, Shuai Xu, Xuan Liu, Xiaolong Zhang, Jing Hu

**Affiliations:** 1 Hubei Key Laboratory of Intelligent Information Processing and Real-Time Industrial System, School of Computer Science and Technology, Wuhan University of Science and Technology, Wuhan 430065, China; xiaolong.zhang@wust.edu.cn (X.Z.); hujing@wust.edu.cn (J.H.); 2 School of Computer Science and Technology, Wuhan University of Science and Technology, Wuhan 430065, China; xushuai@wust.edu.cn (S.X.); lx03068@wust.edu.cn (X.L.)

**Keywords:** drug–target interactions, similarity fusion, graph isomorphic network, TextGNN

## Abstract

**Simple Summary:**

Accurate identification of potential targets for drugs to interact with can accelerate drug development. The identification of drug–target interactions can provide insights into hidden drug efficacy. This paper presents a prediction model based on feature similarity fusion that can identify crucial features of drugs and targets to help predict drug–target interactions.

**Abstract:**

The key to drug discovery is the identification of a target and a corresponding drug compound. Effective identification of drug–target interactions facilitates the development of drug discovery. In this paper, drug similarity and target similarity are considered, and graphical representations are used to extract internal structural information and intermolecular interaction information about drugs and targets. First, drug similarity and target similarity are fused using the similarity network fusion (SNF) method. Then, the graph isomorphic network (GIN) is used to extract the features with information about the internal structure of drug molecules. For target proteins, feature extraction is carried out using TextCNN to efficiently capture the features of target protein sequences. Three different divisions (CVD, CVP, CVT) are used on the standard dataset, and experiments are carried out separately to validate the performance of the model for drug–target interaction prediction. The experimental results show that our method achieves better results on AUC and AUPR. The docking results also show the superiority of the proposed model in predicting drug–target interactions.

## 1. Introduction

The mechanism of drug efficacy involves a drug binding to the relevant site of a target protein and causing a biochemical reaction [1] which affects the biological activity of the protein. The challenge in drug discovery is that the known binding sites for drug–target interactions are not well enough understood [2,3]. The accurate and efficient identification of drug–target interactions can help to reduce the scale of drug screening [4].

With the development of intelligent computing, the speed of drug screening has greatly improved and the cost savings are considerable [5,6]. Deep neural networks can simulate any complex function and have recently been used widely [7,8]. In addition to the different designs of the models, the preprocessing of drug molecule sequences and target protein sequences are also different. With some prediction models, sequences of target proteins and SMILES sequences of drugs are inputted directly into the model [9,10]. Using the extracted feature vector as an input is also a popular approach [11,12,13]. However, local features of drugs or targets may be lost. Wang [14] used a deep learning stacked autoencoder to learn features of the molecular structures of drugs and sequences of target proteins to automatically mine hidden information from protein sequences for the generation of highly representative features. Feature descriptors are also constructed by combining molecular substructure fingerprint information. DeepDTI [15] learns features from drug fingerprints and protein descriptors to achieve predictions of drug–target interactions. DeepDTI mainly focuses on solving the problem of drug repurposing. There are considerable drug–target interactions in the dataset that are unknown, which makes the model prediction biased towards drugs and targets with large numbers of interactions.

The above models usually represent drugs as strings, which is not the natural way to represent molecules. With the use of string representations, some of the structural information of a molecule is lost, which may impair the predictive power of a model and make it difficult to search the whole relevant space [16]. There are still limitations in using only the structural features of drugs and targets to predict drug–target interactions, which depend not only on molecular structure but also on the properties and physiological effects involved in drug–target interactions.

In this paper, an improved prediction model based on the molecular graph representation method is proposed which is combined with the similarity network fusion (SNF) method. The features of internal structural information and intermolecular interaction information for drugs and targets are considered and combined with drug similarity and target similarity. The similarity selection method is used to select subsets of similarity data which contain rich information with low redundancy about drugs and target proteins, respectively. The graph isomorphism network (GIN) is used to extract the structural information of drug molecules, and TextCNN is used to extract the features of target proteins. Drug–drug similarity graphs and target–target similarity graphs are constructed by fusing the similarity datasets. Then, the node features containing inter-molecular similarity information are further extracted using graph convolutional networks. Interaction information for drugs and targets is extracted using graph convolution based on drug–target interaction graphs. Finally, drug–target interactions are predicted by a classifier composed of a multilayer neural network with a fully connected layer and a sigmoid layer. 

## 2. Materials and Methods

### 2.1. Similarity Fusion

Drug similarity is the structural similarity between drugs and drugs, and protein similarity is the similarity in sequence information between proteins and proteins. It is assumed that two drugs or two target proteins can be substituted one for another if there is a high degree of similarity in structure or sequence information. Potential drug–target interactions can also be discovered through topological structure networks, which can make useful contributions to the prediction of potential drug–target interactions.

To obtain more comprehensive features about drugs and target proteins, similarity features are calculated and fused for each drug and each target protein. These features are used to complement each other for building a comprehensive and diverse feature network. Drug similarities and the target similarities are fused before feature extraction is performed on drugs and targets. Given the set of similarity metrics obtained from drugs and target proteins, the goal of similarity fusion is to combine multiple similarity matrices into a composite similarity matrix which can capture the necessary information from the different similarities. Therefore, for drugs and target proteins, sets of multiple similarities are given separately, and the final fusion similarity is calculated according to the similarity network fusion (SNF) method.

Combining all similarity matrices may introduce noise, so it is necessary to first select the suitable subset from the existing similarity dataset. The subset of similarity data is selected using the similarity selection algorithm proposed in [17], which enables the selection of a set of information-rich and less redundant similarity data for drugs and target proteins, respectively. This is achieved through a heuristic process in which a subset of similarity metrics is selected to form an optimal combination of similarities.

The calculation process is as follows.

1. Calculate the average entropy of each similarity matrix to determine how much information each similarity contains.
2. Arrange the matrices in ascending order according to the average entropy value of the matrices. The lower the average entropy value, the less random is the information carried by the similarity matrix. Then, the similarity matrices with high average entropy values are removed.
3. Calculate the similarity measure Es between similar matrices from different data sources according to Euclidean distances.
4. Remove the redundant similarity matrices based on the similarity measure Es between similar matrices.

Based on the obtained subset of drug similarities and target similarities, multiple similarity matrices are combined into a final composite similarity matrix by similarity fusion to capture the necessary information from the different similarities. The similarity network fusion method is based on message passing theory and uses a nonlinear approach to combine multiple similarity matrices into a single fused similarity matrix. It iteratively updates each similarity network with information about other networks and uses k-nearest neighbors to make the current network more similar to other networks. The similarity network fusion method can capture both common and complementary information in different similarity matrices which can be used to integrate multiple drug–drug similarities and target–target similarities, respectively.

### 2.2. Feature Extraction

The most critical issue concerning drug–target interactions is the extraction of features which can effectively characterize targets and drugs. More effective features should be extracted for better prediction of drug–target interactions. In this paper, features are extracted with molecular internal structural information and intermolecular interaction information from drugs and target proteins, with drug similarity and target similarity combined based on graph representations for drugs and targets.

Given a network G=(V, R), $V_{drug}$ and $V_{target}$ are the drug nodes and the target nodes, respectively. $v_{drug}\;\in \;V_{drug}$ and $v_{target}\;\in \;V_{target}$. If the similarity between drug A and drug B is greater than 0.2, which is considered as an edge between drug A and drug B, this edge is noted as $r_{drug}$, and $r_{drug}\;\in \;R_{drug}$. If the similarity between target protein A and target protein B is greater than 0.2, which is considered as an edge between target protein A and target protein B, this edge is noted as $r_{target}$, and $r_{target}\;\in \;R_{target}$. 

The properties of drugs and target proteins are significantly different. If a common feature extraction method is used in drug feature extraction and target feature extraction, the characteristics of drugs and targets are not reflected as well as they might be. Thus, two different models are used to obtain the features of drugs and target proteins.

#### 2.2.1. Feature Extraction of Drugs

The SMILES sequences of drugs can be easily converted into two-dimensional graph structures, and the composition and structural features of drugs can be obtained through graphs. Therefore, this paper uses a feature extraction strategy based on a graph neural network for obtaining the features that most effectively represent a drug. In this work, the graph isomorphic network (GIN) [18] is selected as the main method for extracting drug features. The GIN is an architecture based on graph neural networks. The representational ability of a graph neural network is strengthened by introducing a multi-layer perceptron (MLP). GIN can better identify different structures and capture the dependencies between graph structures. For the input graph G = (V, E), the GIN layer will calculate it as follows:

(1) $$h_{v}^{\left(k\right)}=MLP^{\left(k\right)}\left(\left(1+\epsilon^{\left(k\right)}\right)\cdot h_{v}^{\left(k-1\right)}+\underset{u\in N\left(v\right)}{\sum }h_{u}^{\left(k-1\right)}\right)$$

where k is the number of iterations, ϵ denotes a learnable parameter or a fixed scalar, $h_{v}$ denotes the feature vector of node v, and N(v) is the set of neighbor nodes of node v. 

The SMILES sequence of a drug molecule is encoded as a graph G = (V, E) with feature nodes, where the vertices of the graph represent the set of atoms of drugs. Each atom is represented by a vector consisting of five features (atomic degree, atomic symbol, implicit value of the atom, sum of hydrogens, and aromatic compounds or not). The edges of the graph represent the set of bonds of drugs, which is represented as the adjacency matrix. The steps for converting SMILES sequences to a graph are shown in Algorithm 1.
**Algorithm 1** Converting SMILES sequences to a graph Step 1: Obtain the structure of drug molecules by SMILES sequence;  Step 2: Calculate the number of atoms according to the structure of drug molecule; Step 3: Get the set of atoms according to the structure of drug molecule; Step 4: Obtaining the five features of each atom; Step 5: Five features expressed as one-hot vectors; Step 6: Merge five one-hot vectors to obtain the set of atomic feature vectors; Step 7: Obtain edge sets based on drug molecular structure; Step 8: Get the starting atom and ending atom of the edge; Step 9: Obtain the adjacency matrix from the set of edges.

**Figure 1:**
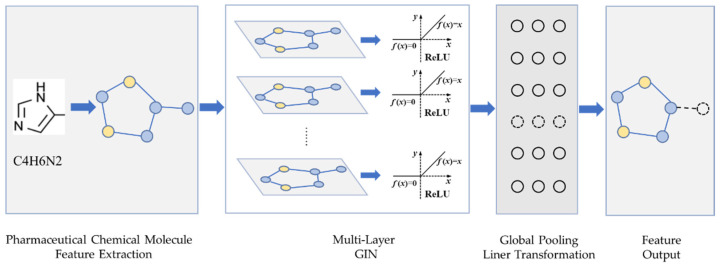
Feature extraction model for drugs.

The drug feature extraction module is shown in Figure 1, where each layer of the GIN units is followed by a ReLU activation layer and a batch normalization layer. The output tensor of the hidden layer is imported into the global pooling layer. The drug feature extraction module can obtain the drug feature vector by the linear transform layer and dropout layer that processes the output tensor of the pooling layer.

#### 2.2.2. Feature Extraction of Target Proteins

For target proteins, TextCNN is used for extracting their features, which has the advantage of capturing local features of input sequences. The core of TextCNN is the convolutional module, which consists of three parts: the input layer for word-embedding operations, the one-dimensional convolutional layer for capturing features, and the pooling layer for the filtering and downscaling of features. 

To extract target protein features, the amino acid sequence of a target protein needs to be represented by a word vector first. The word vector is linearly transformed through the embedding layer and then inputted into the convolution module to obtain the structural features of the target protein. The features obtained are used as node features in the target similarity graph, and further target node features are generated using the GCN. The GCN layer [19] is defined as follows.

(2) $$f{\left(X,A\right)}^{\left(l+1\right)}=T\left(\sigma \left({\tilde{D}}^{-\frac{1}{2}}\tilde{A}{\tilde{D}}^{-\frac{1}{2}}X^{l}W^{l}\right),X\right)$$

where X denotes the feature set, A denotes the adjacency matrix, σ denotes the activation function, $\tilde{A}$ denotes the adjacency matrix with self-looping, $\tilde{D}$ denotes the degree of matrix $\tilde{A}$, and W denotes the random weight. 

#### 2.2.3. Training

The drug–target interaction graph (DTI-Graph) is constructed based on obtained features. Interaction information between drugs and targets is extracted using graph convolution based on the DTI-Graph. Finally, drug–target interactions are predicted by a classifier composed of a multilayer neural network with a fully connected layer and a sigmoid layer.

During the training of the model, the loss function is used to calculate the loss value and the optimization function is used to minimize the loss for continuous optimization of the model. The loss function is used to measure the degree of difference between the predicted value and the true value. The binary cross-entropy loss function and the Adam optimization function are used in this paper. The loss function is calculated as follows.

(3) $$L\left(y,f\left(x\right)\right)=-\frac{1}{N}\overset{N}{\underset{i=1}{\sum }}\left[y_{i}\log P+\left(1-y_{i}\right)\log Q\right]$$

where $y_{i}\in \left\{0,1\right\}$ denotes the true label of the sample and f(x) denotes the predicted outcome of sample x. $P=q(y_{i}=1|x_{i})$ denotes the probability that sample $x_{i}$ belongs to category 1, while Q=1−P denotes the probability that sample $x_{i}$ belongs to category 0.

## 3. Results

### 3.1. Datasets

In this paper, a standard dataset was used to evaluate the predictive performance of the proposed model. This standard dataset was derived from the DrugBank, KEGG, BRENDA, and SuperTarget databases by Yamanishi et al. [20]; it contains a diverse array of target proteins and drugs with rich information. This dataset is divided into four subsets based on the types of target proteins: enzymes (E), ion channels, (IC), G protein-coupled receptors (GPCR), and nuclear receptors (NR). Since its publication, it has been validated and used for a large number of drug–target interaction prediction models, and many models have used it as a standard for validation. We also collected information on the SMILES sequences of drugs and the sequences of target proteins from DrugBank. The similarity measure for drugs and targets was introduced, which was based on the similarity assumption that if drug A has a known interaction with target 1, then drug B, which has high similarity to drug A, may also interact with target 1. For a drug–target pair that is not yet known to interact, the proposed model can find drugs and targets with high similarity to learn features related to them for finding new drug–target interactions. The similarity values of drugs and the similarity values of target proteins were obtained from [21]. The details of the dataset are shown in Table 1; the enzyme dataset removes two targets without amino acid sequences and three sets of interaction information associated with these two targets. 

### 3.2. Evaluation Metrics

In an experiment, the dataset can be divided into positive samples and negative samples. TP means the sample is actually positive and the prediction result is also positive. FN means the sample is actually positive and the prediction result is negative. FP means the sample is actually negative and the prediction result is positive. TN means the sample is actually negative and the prediction result is also negative. The confusion matrix is as shown in Table 2.

Accuracy is one of the most common evaluation metrics and indicates the proportion of samples classified correctly relative to the total number of samples. It is defined as:

(4) $$\mathrm{Accuracy}=\frac{TP+TN}{TP+FP+TN+FN}$$

Precision indicates the percentage of results predicted as positive samples that were predicted correctly. Recall indicates the ratio of samples correctly predicted as positive samples relative to the actual total of positive samples. Precision and Recall are defined as follows:

(5) $$\mathrm{Precision}=\frac{TP}{TP+FP}$$

(6) $$\mathrm{Recall}=\frac{TP}{TP+FN}$$

The PR curve is formed with Recall as the horizontal axis and Precision as the vertical axis, and the area under the PR curve is the AUPR value. AUPR is used as one of the evaluation metrics in this paper. 

In addition, AUC is the most important evaluation metric in the binary classification task and reflects the classifier’s ability to rank the samples. Both TPR and FPR are required to calculate the AUC value. The true positive rate (TPR) is equivalent to Recall and the negative positive rate (FPR) indicates the rate of negative samples predicted incorrectly. The curve formed by TPR and FPR is called the ROC curve, and the area under the ROC curve is the AUC value. The larger the AUC value, the better the performance of the model. TPR and FPR are defined as follows:

(7) $$FPR=\frac{FP}{FP+TN}$$

(8) $$TPR=\frac{TP}{TP+FN}$$

### 3.3. Parameter Setting

In this paper, five-fold cross-validation and retention-type experiments were used to validate the performance of the model. Retention-based experiments divided the dataset according to three different methods: (1) CVD, which divides the dataset according to drugs, where drugs that have appeared in the training set will not appear in the validation set. This division can contribute to new drug development; (2) CVT, which divides the dataset according to target proteins, where target proteins that have appeared in the training set will not appear in the validation set. This can contribute to finding new target proteins for known drugs that prove efficacious; (3) CVP, which divides the dataset according to drug–target pairs, where the drugs and target proteins are known but the interactions between them are not necessarily known. This division can contribute to the discovery of new interactions between known drugs and known targets.

The experiments were conducted using four subsets of the standard dataset. Each training dataset was composed of known positive samples and randomly generated negative samples. Using five-fold cross-validation, five experiments were performed on the same dataset and the final results were averaged. For each training step, 80% of the training set was used for training and 20% for testing.

### 3.4. Analysis of Results

**Figure 2:**
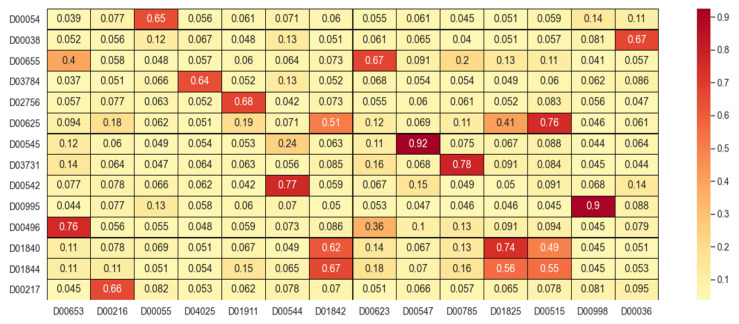
Partial results of similarity fusion.

First, drug similarity and target similarity were converged to form a fused similarity dataset. For each training step, the structural information features of drugs and targets were first extracted from the drug SMILES sequences and target protein sequences. Then, based on the drug composite similarity matrix and target composite similarity matrix obtained from the fused similarity dataset, drug–drug interaction graphs and target–target interaction graphs were constructed. Features containing deep-level interaction information were extracted from drugs and target proteins, respectively. Some results of similarity fusion were visualized in similarity matrixes, as shown in Figure 2. The deeper the color and the higher the saturation, the greater the similarity between samples. 

Then, the DTI-Graph was constructed based on the known drug–target interactions in the training set, and the previously extracted drug–target features were used as node features in the DTI-Graph. The drug–target features were further extracted using graph neural networks. Finally, the drug features and target features were concatenated and inputted into a multilayer neural network classifier for prediction of drug–target interactions.

**Figure 3:**
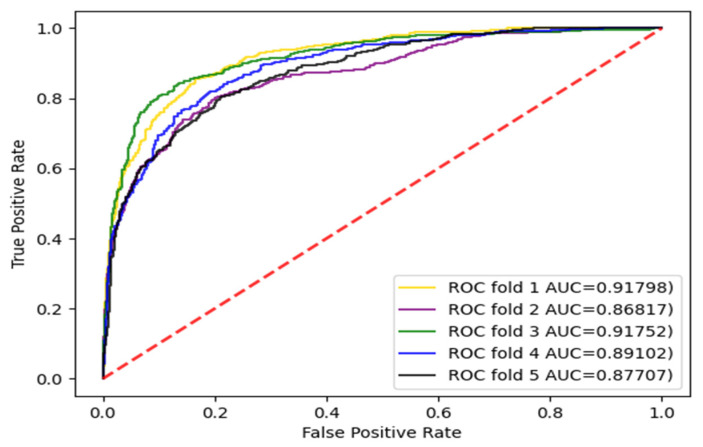
AUC curve of the enzyme dataset under CVD division.

**Figure 4:**
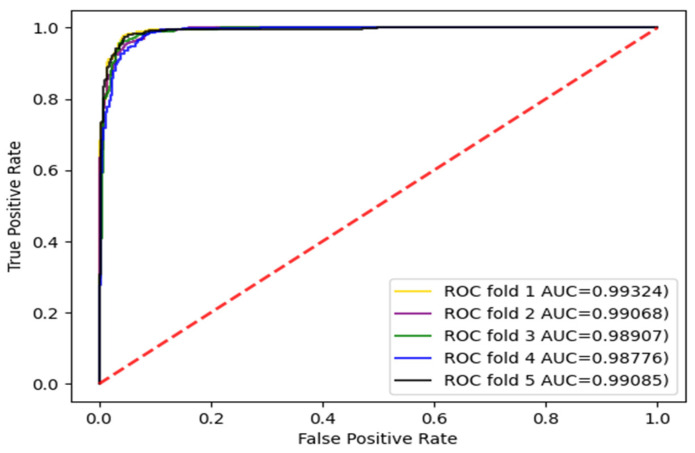
AUC curve of the enzyme dataset under CVP division.

**Figure 5:**
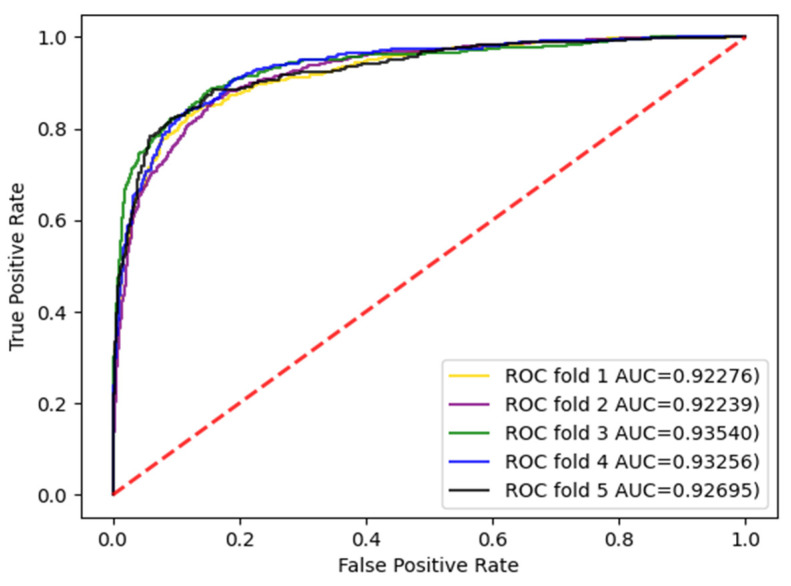
AUC curve of the enzyme dataset under CVT division.

The experiments were carried out with three divisions, respectively, and the dataset was divided into five parts and repeated five times. Each time, a different subset was selected as the test set and the other subsets were selected as training sets. The AUC and AUPR values were calculated. The enzyme dataset was the subset with the largest sample in the standard dataset. Therefore, the AUC curves for the enzyme dataset with three divisions according to CVD, CVP, and CVT were given, as shown in Figure 3, Figure 4 and Figure 5.

**Figure 6:**
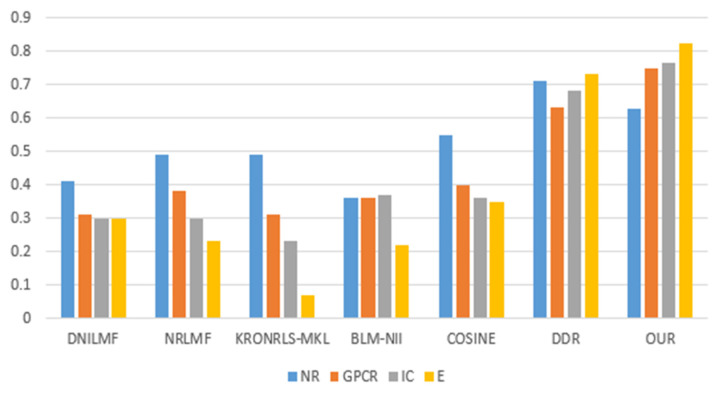
CVD partitioning of experimental results.

**Figure 7:**
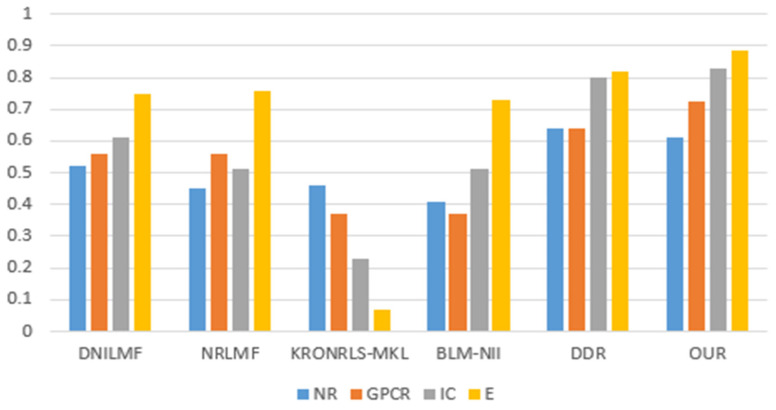
CVT partitioning of experimental results.

**Figure 8:**
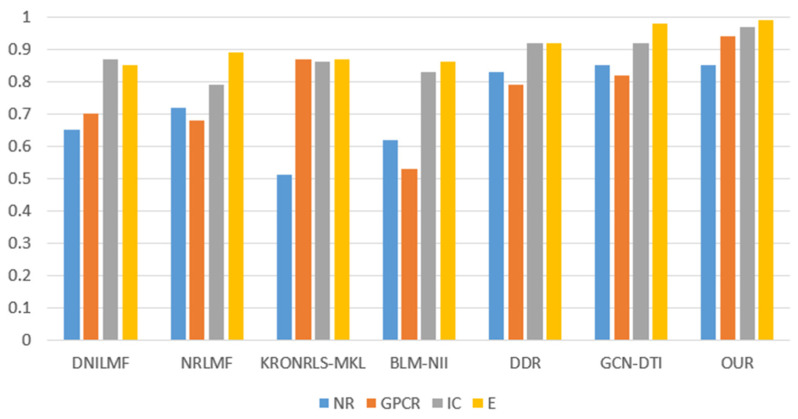
CVP partitioning of experimental results.

For the three divisions, most of the existing methods use AUPR values to evaluate the performance of the methods. Therefore, to make a fair comparison, we also performed comparisons with some existing methods, such as DNILMF [22], NRLMF [23], KRONRLS-MKL [17], BLM-NII [24], COSINE [25], and GCN-DTI [6], in terms of AUPR values. The comparisons of experimental results with CVD, CVT, and CVP divisions are shown in Figure 6, Figure 7 and Figure 8.

By means of the comparisons, it was found that the proposed method is significantly better than the other existing methods; the proposed model is superior in terms of predicting drug–target interactions by representing drug molecules graphically to extract molecular features in combination with drug similarity and target similarity. 

## 4. Discussion

This paper proposes a new method for predicting drug–target interactions that combines similarity fusion and a graph neural network. According to the similarity hypothesis, structurally similar drugs have similar pharmacological effects and structurally similar targets may also have similar biological effects. If drug A interacts with target 1, then drug B, which is highly similar to drug A, may also interact with target 1. The proposed model integrates similarity between drugs, similarity between targets, and known drug–target interactions to predict unknown drug–target interactions. Furthermore, it is possible to use this model to find new similar drugs or new similar targets. 

In addition, in the experiment, the CVD partitioning method helped to predict a new drug. The drugs that appeared in the test set never appeared in the training set. The drugs used in the test set corresponded to new drugs, and all interactions associated with these drugs were unknown. In the CVD partitioning method, the targets in the test set never appeared in the training set. The targets used in the test set corresponded to new targets, and the interactions associated with these targets were all unknown.

In this proposed model, the one-dimensional sequences representing drug molecular structures are converted into graphs and the graph isomorphism network (GIN) is used to extract the structural information of drug molecules. Target protein features are extracted using TextCNN. 

Drug–drug similarity graphs and target–target similarity graphs are constructed by optimally selecting and fusing drug similarity and target similarity, respectively. The drug features and target features extracted are used as node features in the similarity graphs. The node features containing intermolecular similarity information are further extracted using graph convolutional networks.

Then, a DTI-Graph is constructed and the interaction information between drugs and targets is extracted using graph convolution to generate features of drugs and targets. Based on the drug features and target features obtained, drug–target interactions are predicted by a classifier consisting of a multilayer neural network fully connected layer and a sigmoid layer. The fully connected layers can integrate local information with category differentiation. To enhance the network performance, a linear transformation of the network is performed using the ReLU activation function following each fully connected layer, which can increase the nonlinear relationship between each layer of the neural network. The sigmoid layer is able to convert any real number into a probability by compressing it to between 0 and 1. The larger the input value, the more the compression value tends to 1, and the normalized sum is also ensured to be 1.

### 4.1. Performance of the Three Divisions

**Figure 9:**
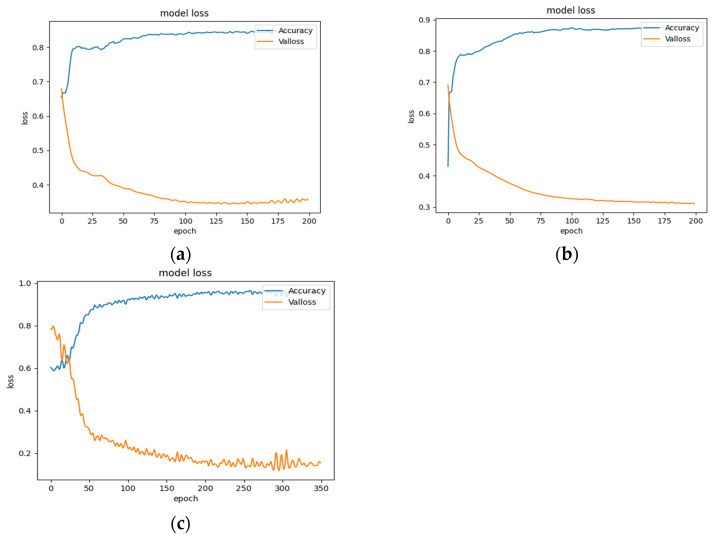
Curves of loss value and accuracy during training. (**a**) CVD. (**b**) CVT. (**c**) CVP.

As an example, with the largest subset of the enzyme dataset in the standard dataset, the change of loss value and accuracy of the test set under the three divisions is shown in Figure 9. It can be shown that the loss values and accuracy of the CVD and CVT divisions gradually level off to a stabilized state when the number of training steps reaches about 200. This indicates that the model has fully learned and reached the optimal value or found a local optimal value. Under the CVP division, the model is trained up to about 300 times before it approaches a stabilized state. This indicates that the model can learn more information under the CVP division.

The experiments were performed on the standard dataset under different division methods, and the AUC and AUPR evaluation metrics were used to measure the model’s prediction performance. The AUC value is the area enclosed with the coordinate axis under the ROC curve, and the ROC curve combines the true positive rate and the false positive rate in a graphical way. It can accurately reflect the relationship between the true positive rate and the false positive rate in the model prediction results. These evaluation metrics are representative of comprehensive detection accuracy. The closer the AUC is to 1.0, the higher the reliability of the model prediction results. AUPR is the area under the curve, with Precision as the vertical axis and Recall as the horizontal axis. The curve is affected by sample distribution and the curves can be used to measure the model’s ability to perform on an unbalanced dataset. Considering AUC and AUPR together is the way to show the generalization ability of the model. 

**Figure 10:**
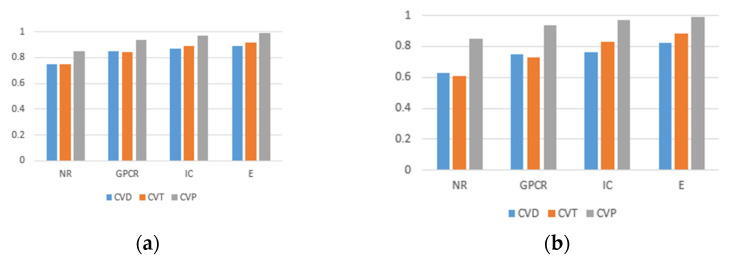
The AUC values and AUPR values of experimental results under three division methods. (**a**) AUC values of prediction results. (**b**) AUPR values of prediction results.

Figure 10 shows the AUC values and AUPR values of prediction results under different division methods. The results of CVP were significantly better than those of CVD and CVT in all three classifications, which was due to the possibility that in the case of classification by drug–target pairs, some of the known interactions between drugs and targets present in the test set were already present in the training set. In addition, the model can extract more features with relevant interaction information, so the prediction results will be significantly improved.

### 4.2. Docking Analysis

The proposed model can also be used to predict potential drug–target interactions related to COVID-19. We collected 366 potential drug–target interactions from the COVID-19 column of DrugBank, including 25 drug molecules and 347 targets. Then, drug IDs, drug names, and SMILES sequences for these 25 drugs were obtained from the DrugBank database. Target IDs, target names, and sequences for these 347 targets were obtained from the PDB database. There were 299 data related to *Fostamatinib* (DB12010) out of 366 potential drug–target interactions. 

Drug–target interactions for COVID-19 were predicted and the drug–target pairs with the top scores were assessed in docking experiments to verify the feasibility of the model. For example, the predicted drug *Fostamatinib* (DB12010) is considered as a potential treatment for the control of patients with acute respiratory distress syndrome (ARDS) in severe COVID-19 by modulating the ability of SYK kinase [26,27]. *Metenkefalin* (DB12668) is an investigational endogenous opioid being studied for the treatment of COVID-19, which is being investigated as an immunomodulatory therapy for moderate or severe patients [28]. *Dexamethasone* (DB01234) has been used in patients with COVID-19 who have severe respiratory symptoms [29].

Docking was performed using AutoDock Vina [30]. RMSD values (root-mean-square deviation of atomic positions) for the conformations of drug molecules were calculated using Pymol. Due to the variation that occurs in the RMSD values for each calculation, the RMSD values that were essentially stable over several calculations were recorded. Table 3 shows the predicted top drug–target docking results and the RMSD values. According to the target’s accession number in UniProt [31], mapped to AlphaFold and RCSB PDB, the corresponding PDB file can be obtained.

The best affinity for docking results of the drug DB12668 and the target P35372 reached −7.7 kcal/mol, which is lower than the −9.5 kcal/mol of the drug DB12668 and the target P41143. The reason for this was that the only information about the target P35372 is the structure predicted by AlphaFold with the prefix ‘AF’ and the known protein information were not abundant.

Since the DrugBank database does not contain the 3D file structures of drug molecules for DB12668 and DB12668, they could only be obtained by transforming the format. From the docking results, it can be seen that the docking results obtained by converting drug molecules from 2D to 3D structures are better only for the target P35372 and the target P41143. Moreover, the lack of 3D structures of known drugs leads to higher RMSD values. The RMSD values of the other docking results for the drug molecules were less than 2. The docking results were relatively reliable, and the affinity of the best docking results was basically less than −7.5 kcal/mol.

For the interaction pairs DB01234 and P04150, DB00959 and P04150, the targets were the same, but the best docking results corresponded to different proteins with different PDB IDs when docked with different drugs. The best docking result between DB01234 and P04150 reached −9.5 kcal/mol, corresponding to PDB ID 3E7C. The best docking result between DB00959 and P04150 reached −8.4 kcal/mol, corresponding to PDB ID 1NHZ.

**Figure 11:**
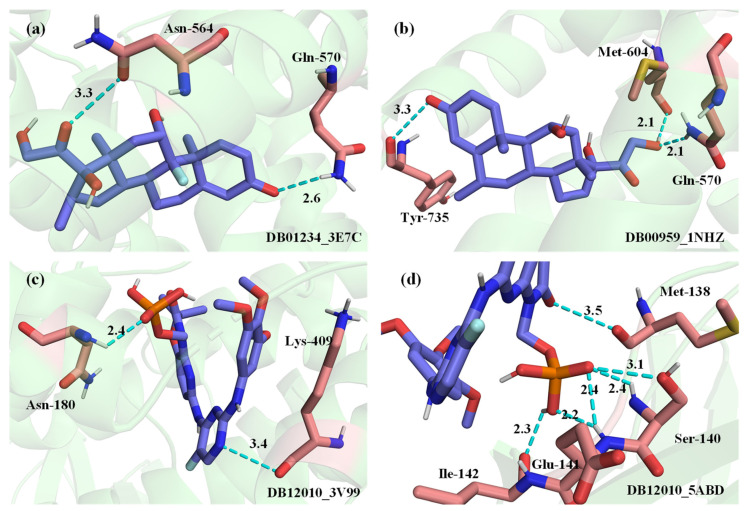
3D structural visualization of docking results. (**a**) DB01234_3E7C. (**b**) DB00959_1NHZ. (**c**) DB12010_3V99. (**d**) DB12010_5ABD.

Four drug–target pairs were selected for visualization, and the docking diagram is shown in Figure 11. The information for the corresponding drug–target pairs is shown in Table 4. In Figure 11, lime green indicates the protein. The drug molecule is presented as purple for carbon atoms, gray for hydrogen atoms, blue for nitrogen atoms, red for oxygen atoms, orange for phosphorus atoms, yellow for sulfur atoms, and dark cyan for iron atoms. The amino acid residues interacting with drug molecules are indicated in pink for the carbon atoms, and the other atoms are indicated in the same color as the drug molecule. The names of amino acids and their locations are identified near the amino acid residues. The polar bonds are indicated by cyan dashed lines. The lower right corner of the figure shows the drug ID and the PDB ID. 

There are still numerous challenges for and limitations to the research program presented here. (1) The known 3D structural information for drugs and proteins is still insufficient. (2) It is also a challenge to select the 3D structure that best represents the protein. The different 3D structures used for docking result in different conformations. In particular, a protein number in the UniProt database usually corresponds to multiple protein 3D structures (different PDB files). There are no uniform criteria for selecting a PDB file. Usually, docking is based on certain empirical strategies for selecting one PDB file. The selection of different PDB files could have an impact on docking analyses. (3) The differences in file format conversion tools and docking tools could also lead to differences in 3D conformations. Facing the above challenges, we will consider further related work based on the similarities of protein fragments combined with relevant type information for ligand molecules.

## 5. Conclusions

This paper proposes a method to predict drug–target interactions by representing drug molecules graphically, combining constructed drug–drug similarity graphs and target–target similarity graphs. In similarity network fusion, a nonlinear approach is used to combine different similarity subsets of drugs and targets to generate the final fused similarities. Three different divisions were used on the standard dataset and experiments were conducted separately to fully validate the performance of the model in predicting drug–target interactions. Using AUC and AUPR values as evaluation metrics, the proposed method achieved better results in comparison with other existing methods. The experimental results showed that the proposed model extracted the features of drugs and targets from multiple perspectives, allowing the obtention of more comprehensive and plentiful features. In addition, the implementation of graphical neural networks makes the model more scalable and superior.

## Figures and Tables

**Table 1:** Statistics on the number of drugs and targets in the standard dataset. There are four subsets: enzymes (E), ion channels (IC), G protein-coupled receptors (GPCR), and nuclear receptors (NR).

Dataset	Drug	Target	Interactions
E	445	662	2923
IC	210	204	1476
GPCR	223	95	635
NR	54	26	90

**Table 2:** Confusion matrix.

	Predicted	Positive Sample	Negative Sample
Actual			
Positive sample		TP	FN
Negative sample		FP	TN

**Table 3:** Docking results for drug–target pairs with the top scores.

Drug ID	Target ID	PDB IDs	Affinity	RMSD
DB12668_web	P35372	AF_P35372	−5.9	3.481
DB12668_obb_2	P35372	AF_P35372	−7.7	3.984
DB12668_obb_s	P35372	AF_P35372	−6.8	1.699
DB12668_web	P41143	6PT2	−8.3	4.680
DB12668_obb_2	P41143	6PT2	−8.8	2.424
DB12668_obb_s	P41143	6PT2	−7.3	2.421
DB12668_web	P41143	6PT2_1	−8.1	3.736
DB12668_obb_2	P41143	6PT2_1	−9.5	2.586
DB12668_obb_s	P41143	6PT2_1	−7.9	3.390
DB01234	P04150	1NHZ	−8.6	0.000
DB01234	P04150	3E7C	−9.5	0.001
DB01234	P04150	3E7C_1	−7.3	0.000
DB01234	P04150	3K22	−7.3	0.000
DB01234	P04150	3K22_1	−7.9	0.000
DB01234	P04150	4MDD	−7.5	0.001
DB01234	P04150	4MDD_1	−7.0	0.000
DB01234	P04150	5E69	−6.9	0.001
DB01234	P04150	5E69_1	−6.9	0.000
DB00959	P04150	1NHZ	−8.4	0.000
DB00959	P04150	3E7C	−7.2	0.000
DB00959	P04150	3E7C_1	−7.4	0.000
DB00959	P04150	3K22	−6.9	0.000
DB00959	P04150	3K22_1	−7.1	0.000
DB00959	P04150	4MDD	−8.2	0.000
DB00959	P04150	4MDD_1	−7.1	0.000
DB00959	P04150	5E69	−7.2	0.000
DB00959	P04150	5E69_1	−7.2	0.001
DB01050	P35354	5F19	−7.5	1.858
DB01050	P23219	6Y3C	−5.7	1.398
DB01050	P23219	6Y3C_1	−5.7	0.759
DB01050	P23219	6Y3C_2	−7.5	0.974
DB01050	P23219	AF_P23219	−5.8	1.418
DB12010	P09917	3O8Y	98.6	0.001
DB12010	P09917	3V99	−8.4	0.001
DB00608	Q9BYF1	6M0J_1	−6.3	1.507
DB12010	P17948	4CL7	−6.6	0.001
DB12010	P17948	5ABD	−8.2	0.048

**Table 4:** Docking results of top four drug–target pairs predicted.

Drugs	Targets	PDB IDs	Affinity	RMSD	Predict
DB01234	P04150	3E7C	−9.5	0.001	0.88697904
DB00959	P04150	1NHZ	−8.4	0.000	0.8678755
DB12010	P09917	3V99	−8.4	0.001	0.7590932
DB12010	P17948	5ABD	−8.2	0.048	0.7105389

## Data Availability

Not applicable.
